# Hyperhemolytic Syndrome Complicating a Delayed Hemolytic Transfusion Reaction due to anti-P1 Alloimmunization, in a Pregnant Woman with HbO-Arab/β-Thalassemia

**DOI:** 10.4084/MJHID.2016.053

**Published:** 2016-10-18

**Authors:** Zoe Bezirgiannidou, Anna Christoforidou, Eftychia Kontekaki, Athanasios G Anastasiadis, Spyros I. Papamichos, Helen Menexidou, Dimitrios Margaritis, Georges Martinis, Elpis Mantadakis

**Affiliations:** 1 Blood Transfusion Centre, University General Hospital of Evros, Alexandroupolis, Greece; 2 Department of Hematology, Democritus University of Thrace Faculty of Medicine, Alexandroupolis, Greece; 3 Department of Pediatrics, Democritus University of Thrace Faculty of Medicine, Alexandroupolis, Greece

## Abstract

**Background:**

Hyperhemolytic Syndrome or Hyperhemolytic Transfusion Reaction (HHTR), a life-threatening subset of Delayed Hemolytic Transfusion Reaction (DHTR) is characterized by destruction of both transfused and autologous erythrocytes evidenced by a fall in post transfusion hemoglobin below the pre-transfusion level.

**Case report:**

We describe a case of DHTR due to anti-P1 alloimmunization manifesting with hyperhemolysis in a 30-year-old Greek Pomak woman with thalassemia intermedia (HbO-Arab/β-thalassemia), during the11^th^ week of her first gestation. She was successfully managed with avoidance of further transfusions and administration of IVIG and corticosteroids.

**Conclusion:**

A high index of suspicion for HHTR is of vital importance among clinicians especially since optimal methods for its prevention and treatment remain yet to be defined. Early recognition of HHTR leading to prompt cessation of additional transfusions and initiation of immunosuppressive treatment can be life-saving, especially in clinical settings where limited therapeutic options are available, such as in pregnancy.

## Introduction

Delayed hemolytic transfusion reactions (DHTRs) represent a well-known complication of transfusions, caused by a secondary immune response to an antigen on the donor’s erythrocytes and leading to the destruction of transfused erythrocytes.^1^ In rare cases, DHTR can manifest with hyperhemolysis, a life-threatening complication.^2^

Hyperhemolysis, also known as Hyperhemolytic Transfusion Reaction (HHTR) is characterized by destruction of both transfused and autologous erythrocytes.^2^ This clinical entity is defined by abrupt onset of accelerated intravascular hemolysis, evidenced by a fall in the post transfusion hemoglobin below the pre-transfusion value, markedly elevated lactate dehydrogenase (LDH), indirect hyperbilirubinemia and hemoglobinuria.^2^,^3^ Of note reticulocytopenia is also present, in contrast to other types of hemolytic reactions, where reticulocytosis is usually notable.^4^ HHTR most commonly occurs in patients with sickle cell disease,^2^,^3^,^5^ while occasionally it has been reported in patients with thalassemia^4^,^6^,^7^ and in individuals without underlying hematologic diseases.^8^,^9^

In thalassemic patients, the alloimmunization rate is approximately 11%.^10^ Anti-P1 alloantibodies, whilst often identified, are rarely reported to trigger hemolytic transfusion reactions.^11^–^13^ Herein, we describe a case of DHTR due to anti-P1 alloimmunization manifesting with HHTR in a 30-year-old pregnant woman with thalassemia intermedia due to compound heterozygosity of HbO-Arab/ β-thalassemia.

To our knowledge, this is the first case of HHTR in a patient with HbO-Arab/β thalassemia. Our report emphasizes the need for the thorough investigation and proper management of HHTR in pregnancy, where limited therapeutic choices are available.

## Case Report

**Figure 1:**
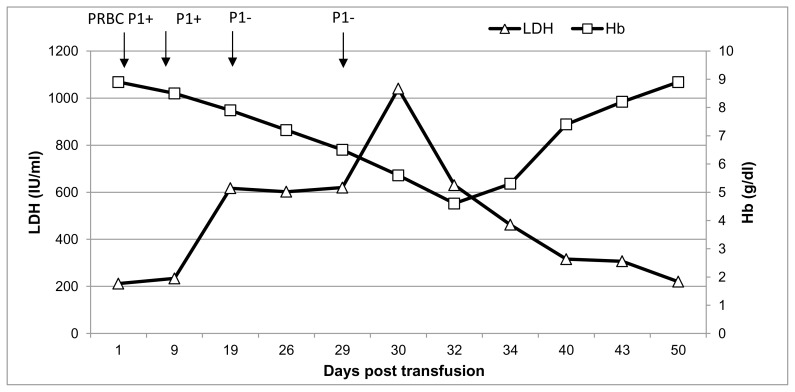
Serial changes in hemoglobin and LDH from d1 to d50. The timing of erythrocyte transfusions and the onset of DHTR [IAT+] are also shown. IVIG: Intravenous immunoglobulin, MP: Methylprendisolone.

A not previously transfused 30-year-old Greek Pomak woman with thalassemia intermedia due to compound heterozygosity for HbO-Arab/β-thalassemia was scheduled for transfusion of erythrocytes during the 11^th^ week of her first gestation. Erythrocytes of the patient and her husband were thoroughly typed for antigens in the main blood group systems, including ABO, Rh, Kell, Kidd, Duffy, MNS, Lewis, Lutheran and P1, in order to avoid the onset of hemolytic disease of the fetus and newborn (HDFN) due to alloimmunization. The blood group phenotypes of the patient and her husband are shown in Table 1. Initial standard screening with direct antiglobulin test (DAT) and indirect antiglobulin test (IAT) was negative. Our initial decision was to transfuse C-, Fy (b-), P1- leukoreduced erythrocytes; however blood units bearing such a seldom antigen combination are usually unavailable in our blood bank. Thus, we decided not to consider P1 antigen in our phenotypically-matched transfusion choice because it is uncommon for an anti-P1 antibody to cause hemolytic transfusion reactions.^11^–^13^ Moreover, anti-P1 is an IgM class antibody that is highly unlikely to cross the placenta and to affect the fetus.^11^ Pre-transfusion hemoglobin level on day 1 (d1) was 8.9 g/dl (Figure 1) and the reticulocyte count was 5.67% (corrected count 1.75%), while LDH was 212 IU/L (normal range<246 IU/L). The patient received the first erythrocyte transfusion without any adverse effects. On d9 she received two more compatible units. On d19, during the next pre-transfusion testing, we observed that the patient’s erythrocytes yielded a positive DAT against anti-C3d [1+] and a positive IAT against P1+ erythrocytes at 37°C, 22°C and 4°C, signifying the presence of an anti-P1 alloantibody. Laboratory findings included hemoglobin (7.9 g/dl), haptoglobin (8 mg/dl), LDH (617 IU/L) and indirect bilirubin (IB) (1.2mg/dl), denoting the onset of a DHTR. Therefore, we decided to transfuse the patient with a C-, Fy(b-), P1- unit. On d29 the patient was admitted to the Department of Hematology because of reddish urine. Laboratory testing revealed hemoglobinuria without erythrocyturia. The hemoglobin had dropped to 6.5 g/dl, while LDH was 620 IU/L and IB was 2.4 mg/dl. Subsequent standard immunohematological screening revealed subtle changes, namely a 2+ positive DAT against anti-C3d. IAT remained positive due to the presence of an anti-P1 alloantibody and cross-match was compatible with P1- erythrocytes. Accordingly, taking into account the IAT and DAT findings as nearly stable, continuing transfusions of erythrocytes were deemed necessary. Hence, the patient received a C-, Fy(b-), P1- unit. Whilst a transient post-transfusion increase in the hemoglobin level was documented on d29 (8.3 g/dl), on d30 the hemoglobin had markedly dropped to 5.6 g/dl, while both LDH and IB were highly elevated (1,040 IU/L and 7.9 mg/dl, respectively). At this point, hyperhemolysis was immediately considered leading to prompt cessation of further transfusions.

We performed an extensive immunohematologic screening using a gel microcolumn system (BIORAD, Cressier sur Morat, Swiss).^14^ Acid elution and cold acid elution (DiaCidel®/BIO-RAD) were performed on the patient’s DAT-positive erythrocytes. The eluate was screened by IAT against a commercially available panel of erythrocytes (ID-DiaPanel) using Liss/Coombs gel cards with polyspecific antihuman globulin (PAHG) as well as DC-Screening I gel cards with monospecific antihuman globulin MAHG (anti-IgG, anti-IgA, anti-IgM)]. Whilst acid elution IAT did not reveal any warm autoantibodies, cold acid elution IAT was positive, yielding 4+ reactivity with PAHG cards and 2+ reactivity with anti-IgM MAHG cards. Following treatment with 1,4-dithiothreitol (DTT®-Sigma Aldrich, USA),^14^ the cold acid elution IAT was negative, both in Liss and DC-screening cards, a finding denoting the presence of a cold-type IgM class autoantibody. Further investigation with cold agglutinin titration revealed anti-I specificity, a finding further reinforced by the complete adsorption of the autoantibody by Rabbit Erythrocyte Stroma (RESt^TM^-Immucor, USA). Autohemolysis during storage signified complement activation. Detection test for isoagglutinins by heat elution and Donath Landsteiner test were both negative excluding from the differential diagnosis passenger lymphocyte syndrome and paroxysmal cold hemoglobinuria (PCH), respectively. ID-PNH Test (BIORAD, Cressier sur Morat, Swiss) for paroxysmal nocturnal hemoglobinuria was also negative. Serological studies for EBV, CMV, HIV, HBV, HCV, *Toxoplasma gondii*, *Mycoplasma pneumoniae* and Parvovirus B19 were negative. Further testing for antinuclear and anti*-*dsDNA antibodies was also negative.

After reviewing the medical literature and considering any feto-maternal complications that could occur, we decided to treat the patient with oral methylprednisolone and intravenous immunoglobulin (IVIG).^15^ Hence, she received on d30 IVIG 1g/kg for two consecutive days and was started on oral methylprednisolone 1mg/kg/day. On d32, the hemoglobin continued to drop to 4.6 g/dl, while the reticulocyte count was 4.71% (corrected count 0.8%) (Figure 1). Following treatment initiation, the hemoglobinuria resolved within 5 days [d35], the hemoglobin gradually increased from 4.6 g/dl to 8.2 g/dl in 13 days (d43) and the LDH completely normalized in 20 days (d50). After 7 weeks of continuous methylprednisolone therapy, the dose was gradually tapered to 4mg *per os* every other day. At this dose, the patient showed no detectable signs of hemolysis throughout pregnancy keeping stable hemoglobin between 8.5 g/dl and 9 g/dl. After three months of methylprednisolone therapy, the DAT became negative, the cold agglutinin was undetectable, while IAT remained weakly positive due to the anti-P1 alloantibody. Six months later, both DAT and IAT were negative. The remaining of the pregnancy and the delivery were uneventful, and a healthy neonate P1-, with negative DAT was born.

## Discussion

HbO-Arab results from a single amino acid substitution in the β chain of hemoglobin A.^16^ It is most commonly found in the Balkan Region and the Middle East. In Greece, the mutation seems to have originated in the Pomak population of Thrace.^16^ An HbO carrier usually has normal hemoglobin, while HbO Arab homozygosity is associated with a mild to moderate anemia.^16^ While compound heterozygosity for HbS/HbO-Arab has the clinical course of severe sickle cell disease, compound heterozygosity for HbO/β-thalassemia has the phenotype of thalassemia intermedia. To the best of our knowledge, this is the first described case of HHTR in a patient with HbO/β-thalassemia.

A series of theories have been proposed in order to explain the hemolytic destruction of both allogeneic and autologous erythrocytes in HHTR, including inordinate complete complement activation, phagocytosis by hyperactivated macrophages of erythrocytes covered with C3b in a setting of incomplete complement activation, and HLA antigen-alloantibody reactions.^2^,^17^ In addition, accumulating evidence suggests a complex interplay between autoimmunization and alloimmunization, denoting a non-negligible risk of autoimmunization and AIHA development as a result of alloantibody formation following an allogenic erythrocyte transfusion.^2^,^18^ This phenomenon defines, among others, a condition termed bystander immune hemolysis. The proposed rationale for its occurrence is that alloantibody binding to transfused erythrocytes triggers conformational changes in antigenic epitopes that subsequently stimulate the inordinate production of an autoantibody and/or generates large amount of activated complement components leading to direct erythrocyte lysis.^2^,^18^

Our extensive screening revealed the presence of a cold-type IgM class autoantibody with anti-I specificity. This finding validates that our patient suffered bystander immune hemolysis, manifesting as a DHTR followed by a cold agglutinin syndrome. Generation of the initially detected anti-P1 was likely the triggering event, providing a background for further complement activation and autoantibody production. It is of note that anti-I can also activate complement by a specific antigen-antibody reaction.^19^ A tantalizing hypothesis is that both antibodies activated large amounts of complement, leading synergistically to bystander hemolysis. It is of note that both anti-P1 and anti-I are directed against antigenic epitopes of similar structural characteristics, signifying that anti-I production could have occurred via “epitope spreading”.^20^

The P system, especially the p phenotype has been related with early abortions due to naturally occurring IgG subclass anti-P1PP^k^(antiTj^a^) likely due to reaction of the antibody with the P antigen present on the placenta tissue as well.^21^,^22^ Our patient had P^2^(P1-) phenotype without natural anti-P1, anti-P or anti-P1PP^k^(antiTj^a^) and the alloantibody detected after transfusion, was reacting only with P1+ erythrocytes and not with P1- RBCs, excluding the case of the rare p phenotype. To the best of our knowledge, this is the first reported case where anti-P1 alloimmunization during pregnancy precedes a HHTR. A previously described case of anti-P1alloimmunization in a pregnant woman was complicated with an acute rather than delayed hemolytic transfusion reaction.^11^

Optimal treatment of HHTR has not been defined. Besides that, pregnancy represents a clinical setting in which limited therapeutic options are safe. To this extend, corticosteroids, IVIG and cyclosporine were previously successfully used in both of the reported cases of HHRT that occurred during pregnancy, one in a patient with sickle cell disease 23 and one in a patient with δβ-thalassemia.^24^

HHTR represents a delayed complication of transfusion and alloimunization. It is important to follow the international guidelines of transfusion in order to prevent alloimunization mainly in chronically transfused patients and in pregnant women. A good practice would be to test RBCs for ABO, Rh, Kell, Duffy, Kidd, MNS and P antigens before transfusion therapy, and administer RBCs phenotypically matched at least for C, E, and Kell in addition to ABO and Rh. Moreover in thalassemic pregnant women, it is useful to avoid transfusing antigens absent on the maternal erythrocytes that are present on paternal RBCs to minimize the possibility of HDFN.

In conclusion, a high index of suspicion for HHTR is of vital importance among clinicians treating patients with hemoglobinopathies. In such cases, the immediate cessation of additional transfusions is required for a favorable clinical outcome.

## Figures and Tables

**Table 1:** Blood group phenotypes of the patient and her husband.

Antigen	ABO	Rh[D]	C	c	E	e	Cw	Kell	k	Kpa	Kpb	Fya
Patient	AB	+	−	+	+	+	−	−	+	−	+	+
Husband	B	+	−	+	+	+	−	−	+	−	+	+
**Antigen**	**Fyb**	**Jka**	**Jkb**	**Lea**	**Leb**	**P1**	**M**	**N**	**S**	**s**	**Lua**	**Lub**
Patient	−	+	+	−	+	−	−	+	−	+	−	+
Husband	+	−	+	−	+	+	−	+	−	+	−	+
